# Need assessment for HIV drug resistance testing and landscape of current and future technologies in low- and middle-income countries

**DOI:** 10.1371/journal.pgph.0001948

**Published:** 2023-10-18

**Authors:** Neil Parkin, P. Richard Harrigan, Seth Inzaule, Silvia Bertagnolio

**Affiliations:** 1 Data First Consulting, Sebastopol, CA, United States of America; 2 Department of Medicine, University of British Columbia, Vancouver, BC, Canada; 3 Amsterdam Institute for Global Health and Development, and Department of Global Health, Amsterdam UMC, University of Amsterdam, Amsterdam, The Netherlands; 4 World Health Organization, Geneva, Switzerland; University of KwaZulu-Natal College of Health Sciences, SOUTH AFRICA

## Abstract

Resistance to antiretroviral drugs used to treat HIV is an important and evolving concern, particularly in low- and middle-income countries (LMICs) which have been impacted to the greatest extent by the HIV pandemic. Efforts to monitor the emergence and transmission of resistance over the past decade have shown that drug resistance–especially to the nucleoside analogue and non-nucleoside reverse transcriptase inhibitors–can (and have) increased to levels that can jeopardize the efficacy of available treatment options at the population level. The global shift to integrase-based regimens as the preferred first-line therapy as well as technological advancements in the methods for detecting resistance have had an impact in broadening and diversifying the landscape of and use case for HIV drug resistance testing. This review estimates the potential demand for HIV drug resistance tests, and surveys current testing methodologies, with a focus on their application in LMICs.

## Introduction

Shortly after the first antiretroviral (ARV) drugs were developed, it was discovered that human immunodeficiency virus type 1 (HIV-1) drug resistance (HIVDR) could evolve quickly both in vitro and in vivo, reducing the effectiveness of monotherapy and early dual combination therapies [1, 2]. The individual risk of HIVDR is reduced with combination antiretroviral therapies, but since the start of the global scale-up of antiretroviral therapy (ART) in the early 2000s, levels of HIVDR have been increasing steadily [3, 4]. There is a high prevalence of pre-treatment drug resistance to non-nucleoside reverse transcriptase (RT) inhibitors (NNRTIs) among people initiating first-line therapy in most of the low- and middle-income countries (LMICs) monitoring and reporting resistance data [5]. These levels are even higher in certain subpopulations, including children [5, 6], with pre-treatment prevalence of resistance as high as 68% in some countries. Resistance to antiretrovirals brings great human and economic costs. In 2017, lack of action to address high levels of pre-treatment HIVDR to NNRTIs in sub-Saharan Africa was predicted to result in high death toll and significant increase in both new HIV infections and ART program costs in by 2030 [7].

Different approaches have been used to detect HIVDR including phenotypic and genotypic tests. The most intuitive way to test for HIVDR is to culture the virus in the presence of increasing drug concentration to determine the 50% inhibitory concentration (IC_50_), in an analogous fashion to susceptibility tests for most antimicrobials [8] or antifungals [9]. However, issues of cost, biosafety, sensitivity, and inherent variability have made traditional intact virus phenotypic testing impractical for routine use. Recombinant virus phenotypic assays, which utilize the viral genomic regions of interest (i.e., containing the coding sequences for targets of ARV drugs) from the infected individual in a constant “wild-type” virus vector, have enabled large-scale susceptibility testing [10, 11], at the expense of excluding the potential impact of variation outside the target regions. Additionally, if the constant “wild-type” background is extremely divergent from the patient-derived virus, it is possible that important indirect interactions might be missed. These phenotypic susceptibility tests remain critical to understanding resistance, but require highly specialized and centralized laboratory facilities, and ultimately have not been broadly transferrable to LMICs.

More commonly, indirect tests that rely on the sequence-based detection of mutations associated with HIVDR are used to infer resistance to ARV drugs [12]. Prediction of resistance from genotyping assays is thus dependent on interpretation algorithms, which are derived from associations between sequence variation and drug exposure, phenotypic susceptibility, and/or clinical outcome data [13]. The availability of algorithms to interpret sequence results, and the comparatively advantageous cost of sequencing compared to phenotypic tests, has resulted in a rapid uptake of genotype testing in many countries. While interpretive algorithms for nucleoside RT inhibitors (NRTIs), NNRTIs and protease inhibitors (PIs) are robust and built upon large datasets, the relatively small number of individuals who have failed ART containing integrase (IN) strand transfer inhibitors (INSTIs), especially in LMICs where non-B HIV-1 subtypes predominate [14], make the interpretive algorithms for INSTIs much less robust or complete.

The potential applications of HIVDR testing include surveillance, research, or individual patient clinical management. While HIVDR testing for surveillance is broadly used in LMICs, the cost and complexity of the test has hampered its applicability for individual clinical management. However, new technologies offering the potential for reduced cost per test, greater simplicity and/or near point of care implementation could be important breakthroughs in the near future. Large amounts of HIVDR testing data can also be useful for the development of computational models for prediction of treatment outcomes without genotypic test results because of restricted availability of laboratory testing in LMICs [15].

Several reviews on resistance testing and sequencing technologies in LMICs have been published previously [16, 17]. However, since these publications, treatment paradigms have transitioned to new ARV regimens based on INSTIs instead of NNRTIs, and changes in some of the underlying testing technologies have occurred. Metzner [17] has published a particularly relevant recent review with a focus on the requirements for an optimal genotypic HIV DR assay based on NGS sequencing.

The aim of this review is to provide a description of the potential demand for HIVDR tests in LMICs, to summarize the characteristics (including regulatory status), advantages and limitations of available HIVDR testing strategies, and to describe promising technologies in development. The review focuses on HIVDR testing relevant for commonly used ARV drug classes (NRTIs and NNRTIs, PIs, and INSTIs) and on mutations which occur in the related target regions of the HIV-1 pol gene.

## Methods

To estimate the potential number of HIVDR tests required in LMIC, we relied on two use case descriptions recently developed by WHO [18]. The first use case applies to patients failing ART regimens including an INSTI (likely to be predominantly dolutegravir, DTG). This includes patients who may have had prior treatment experience with NNRTI-based regimens, and children who may have initiated ART with a PI/r- based regimen but switched to abacavir/lamivudine/DTG for reasons other than treatment failure. The second use case is for more treatment-experienced patients, such as those with a history of treatment failure of regimens including an INSTI or an NNRTI as well as a regimen including a PI/r. Historically, this corresponds to “second line” failures in adults, with DR test results being used to optimize third-line ART. It also includes children with prior exposure to NNRTI-based prevention interventions (in the mother or the child) who subsequently fail a boosted protease inhibitor (PI/r)-based regimen. In both use cases, the results from the test can be used to identify patients most likely to benefit from a treatment change (i.e., those with detectable resistance), and to design the optimal next regimen.

The number of people on ART in LMIC, and the proportions with suppressed viral load, were obtained from UNAIDS (https://aidsinfo.unaids.org). The proportion of treated patients receiving DTG and the number of patients receiving PI/r-based second line ART were obtained from the Clinton Health Access Initiative (CHAI) 2022 HIV Market Report [20]. Other sources of information involved in the calculations are described below. Calculations were performed for adults (>15 years old) and children (0–14 years old) separately, since estimates of the proportion on DTG and proportion with elevated viral load are different. Numbers were rounded to the nearest 100 for simplicity. Calculations and assumptions about numbers and proportions of patients meeting use case criteria are those of the authors, and do not represent the views or positions of WHO or CHAI.

To describe the landscape of HIVDR tests, either available or in advanced development, we used a mixed-method approach including a review of (i) published literature (PubMed) and pre-prints (Google Scholar), (ii) abstracts presented at scientific meetings which appeared in Google Scholar since 2015 (iii) diagnostic company websites (including Roche, Abbott, Cepheid, Vela, LabCorp, Quest Diagnostics, Advanced Biological Laboratories, ThermoFisher, Illumina, Oxford Nanopore, Pacific Biosciences, and Aldatu), and (iv) unscripted key informant one-on-one interviews with known developers of HIVDR test kits to explore their research and development plans. Information was gathered between June 2021 and August 2022.

The types of HIVDR tests covered were categorized as follows: (1) sequencing of “bulk” DNAs produced by RT-PCR amplification (including Sanger and dye-primer sequencing); (2) “next generation” sequencing; and (3) point mutation assays. Tests were further grouped within each category based on their regulatory status: approved by US Food and Drug Administration (FDA) or conformité européenne in vitro diagnostic (CE-IVD), research use only (RUO), and “in-house” or lab-developed tests.

## Findings

### HIVDR testing in low- and middle-income countries: A market assessment

#### Potential market size for use case 1

**Fig 1:**
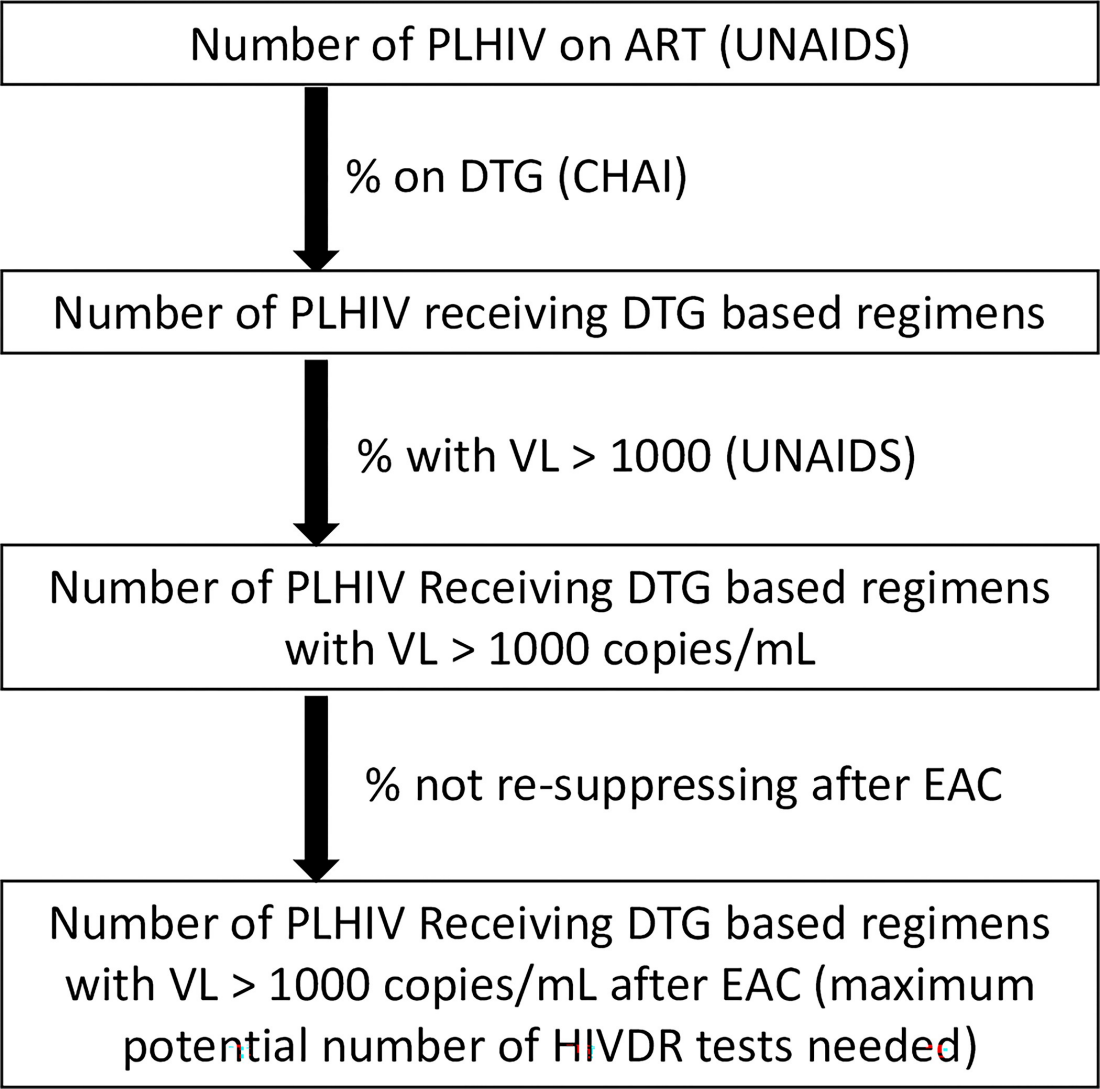
Overview of the process followed to estimate the maximum potential market sized for HIVDR testing in LMIC (use case 1). PLHIV: people living with HIV. ART: antiretroviral therapy. DTG: dolutegravir. CHAI: Clinton Health Access Initiative. EAC: enhanced adherence counselling.

Using estimates of numbers of people living with HIV (PLHIV) who are receiving ART, an approximation of the percentage of ART regimens that include DTG and of people with viral load over 1000 copies/mL, we calculated the number of people receiving DTG with elevated viral load who might be considered eligible for DR testing (Fig 1). Before a switch in ART regimen is undertaken in LMICs, patients are recommended to be assessed for adherence (often through a package of interventions referred to as enhanced adherence counselling or EAC). Thus, the last component of the DR testing market size is the proportion of DTG-treated patients with viral load over 1000 copies/mL who do not re-suppress after EAC. Very few data have been published in this regard. One post-hoc analysis of patients receiving DTG with VL > 1000 copies/mL after 48 weeks of treatment reported that 12 of 15 (80%) had VL < 1000 copies/mL after adherence counselling [19]. This parameter is likely to change with further research and monitoring, and so the calculations below are subject to revision when additional information is available. Using this preliminary estimate of 20% not re-suppressing, we were able to calculate the numbers of patients meeting the WHO definition of treatment failure in each geographic region for 2021 (Table 1). Globally, the number of patients with confirmed treatment failure as defined by WHO is approximately 2% of the number on ART. In subsequent years, it is likely that the number of PLHIV on ART, as well as the percentage on DTG, will increase. From 2018 to 2021, the number of people on ART increased by an average of between 5 and 10% per year in different regions. The percentage of adults on DTG is expected to rise to 88% in 2023 [20]. The percentage of children on DTG is expected to increase rapidly in the coming years, possibly reaching over 90% by 2024 [20]. These two increases, and a projected increase in the total number of PLHIV on ART, would lead to a 36% increase in the total number with confirmed treatment failure compared to 2021.

**Table 1:** Maximum market size for use case 1 (DTG failures).

Geographic Region	N on ART in 2021 ^a^	N on DTG ^b^	N with VL > 1000 ^c^	N (%) with confirmed treatment failure ^d^
Asia and the Pacific	3,998,000	3,194,300	290,700	58,200 (1.5%)
Caribbean	234,700	188,000	24,600	4,900 (2.1%)
East and Southern Africa	16,190,000	12,848,400	914,200	182,800 (1.1%)
Eastern Europe and Central Asia	939,000	752,000	45,500	9,100 (1.0%)
Latin America	1,513,000	1,219,700	110,200	22,100 (1.5%)
Middle East and North Africa	87,700	69,300	7,800	1,600 (1.8%)
West and Central Africa	3,850,000	3,051,000	372,600	74,500 (1.9%)
**All LMIC**	**26,812,400**	**21,322,700**	**1,765,600**	**353,200 (1.3%)**

^a^ Numbers of adults (≥15 years old) and children (0–14 years old) on ART in in SSA in 2021 were obtained from UNAIDS AIDSinfo (https://aidsinfo.unaids.org).

^b^ CHAI estimates that 81% of all adults and 36% of children on ART in LMIC were on DTG in 2021 [20].

^c^ VL suppression rate (<1000 copies/mL) in adults was obtained from UNAIDS AIDSinfo (https://aidsinfo.unaids.org). The failure rate in children was estimated to be twice that in adults. In 2022, approximately 15% of children were failing ART in PEPFAR countries.

^d^ assumes that 20% of people with an initial VL > 1000 will not re-suppress after adherence interventions

#### Potential market size for use case 2

To approximate the number of people who meet this use case definition, the estimated number of adults on second line ART, and the total number of children on ART, were multiplied by the estimated proportion using PI/r-based regimens (Table 2). Since the treatment landscape is evolving, it is challenging to predict how these numbers and proportions will change over time. For example, since initial ART is likely to be based on DTG and the confirmed failure rate is expected to be much lower than historical averages from experience with NNRTI-based ART, the proportion of patients on second line ART should decrease over time. At the same time, the absolute number of patients on DTG is likely to increase. Also, since patients failing an NNRTI-based regimen will likely be prescribed DTG, the proportion of regimens that are based on PI/r should decrease. Given the very large uncertainty about trends over time, no attempt has been made to project the numbers for Use Case 2 beyond what it might have been in 2021 (Table 2).

**Table 2:** Maximum market size for use case 2 (PI/r failures).

	Adults	Children
N on 2^nd^ line (adults)^a^ or on ART (children)^b^	1,330,000	882,800
N on PI/r-based ART ^c^	837,900	414,900
N with VL > 1000 ^d^	125,700	62,200
N (%) with confirmed treatment failure ^e^	61,600	42,900
**Total**		**104,500**

^a^ Numbers of adults [20] and children (https://aidsinfo.unaids.org) on ART in 2021.

^c^ CHAI estimates that 63% of all adults and 47% of children were on PI/r in 2021 [20].

^d^ assumes 85% suppress viral load below 1000 copies/mL

^e^ assumes that 51% of adults, and 31% of children, with an initial VL > 1000 will re-suppress after adherence intervention

Combining the estimated maximum market size for both use cases gives a total of 457,700 tests per year. However, it should be noted that the specifications for the test used may differ between use cases, for example based on whether or not the protease (PR) region is included.

## Methods of testing for HIV drug resistance

### Bulk sequencing

Historically, the mainstay of HIVDR testing has been Sanger-based sequencing methods, as well as dye-primer approaches, which were first developed and validated “in-house” by academic research groups and subsequently adapted into kits for sale by commercial entities. This approach involves the amplification of viral RNA mainly from a patient plasma sample to produce a population of DNA products reflecting the regions of interest from the viral population in the specimen. The sequencing of this “bulk” viral population is then performed through the use of dye-labeled terminating dideoxy-nucleotides (Sanger sequencing) [21] or dye-labeled primers [22].

#### TRUGENE

The Visible Genetics TRUGENE test kit was approved by the US FDA in April 2002. It was based on the use of dye-labeled primers and consisted of the reagents, hardware, and software necessary to generate test results. This was a milestone in HIV resistance testing, because its approval included evidence from randomized clinical trials that there was both short term [23] and longer term [24] benefit to patients from using genotype guided treatment decisions. However, following commercialization by the original developer Visible Genetics, as well as licensees Bayer and Siemens, production of the test was discontinued in 2014.

#### ViroSeq

The Applied Biosystems (ABI) ViroSeq kit was approved by the US FDA in December 2002. It included viral nucleic acid extraction, amplification, and sequencing reagents and interpretation software [25]. The kit was validated for use on ABI 3130/3130xl automated sequencers made by ABI. ViroSeq was discontinued in late 2021, concomitant with the end of the manufacturer’s support for the ABI 3130/3130xl instrument. To date, no replacement product for ViroSeq has been produced, and the current manufacturer (Abbott) has not disclosed whether it will develop alternative HIV drug resistance tests.

#### HIV-1 genotyping kit

Thermo Fisher offers an HIV genotyping kit on a RUO basis; that is, not for individual patient management. The kit is based upon an assay developed at the US Centres for Disease Control [26], and is progressively becoming relatively popular in LMICs. **The** genotyping kit incorporates manufacturing standardization and product quality control. Starting from extracted HIV RNA, the kit includes modules for RT-PCR of the pol gene and sequencing on one of the Thermo Fisher sequencing platforms (Table 3). While the kit itself stops at the generation of raw sequence data, the company recommends basecalling with the Exatype [27] (Hyrax Biosciences) or ReCall [28] software to generate a consensus sequence, followed by HIVDR interpretation with the Stanford HIVdb algorithm (also integrated into the Exatype software). Use of this kit requires users to develop and validate their own RNA extraction procedures. The original kit encompassed codons 6–99 in the PR region and codons 1–251 in the RT region of the pol gene. Both plasma and dried blood spots were evaluated, considerably extending the utility of the assay in LMIC. In mid-2022, a new version of the genotyping kit including IN became available.

**Table 3:** Commercially available bulk/Sanger-based sequencing assays.

	ThermoFisher	Advanced Biological Laboratories (ABL)
Product Name	Applied Biosystems HIV-1 Genotyping Kit with Integrase	DeepChek Assay PROTEASE / REVERSE TRANSCRIPTASE and DeepChek Assay INTEGRASE Genotyping and Drug Resistance
Target region	PR codons 6–99, RT codons 1–251, IN codons 1–288	PR codons 1–99, RT codons 1–320, IN codons 20–280
HIV-1 subtypes	Major HIV-group M viruses	Major HIV-group M viruses
Sample types	Plasma and DBS	Plasma
Assay description	RT-PCR, nested PCR, and sequencing; separately for PR-RT and IN	RT-PCR, nested PCR, and sequencing; separately for PR-RT and IN
Extraction method	User-based	User-based
Time to results	16–24 hours*	~17 hours*
Core equipment	ABI genetic analyzer series	ABI genetic analyzer series
Technical skills required	High	High
Regulatory approval	None, research use only	CE-marked
Strength	Low cost per test, commercially available kit, widely evaluated, in use in many settings, sample extraction using existing platforms, validated with DBS with satisfactory performance	kit-based, commercially available
Weakness	High instrument and maintenance cost, need for high technical skills	High instrument and maintenance cost, need for high technical skills
Availability	Available as a kit from ThermoFisher Scientific	Available as a kit from ABL

* Excludes extraction time which is dependent on the type of method used by each lab

#### Other potential developers

Other test developers with potential interest in this area include Roche Molecular Systems and Cepheid. Roche, however, confirmed that it does not have short term plans to develop HIV drug resistance tests, and Cepheid had not yet responded by the time this report was written.

#### Lab-developed genotypic tests

Generally, tests performed in research laboratories do not have regulatory approval for use in individual patient management in North America or Europe. As such, they are usually less expensive and more adaptable than kit-based sequencing methods, but inherently lack some of the standardization and quality control that manufactured kits provide. Some laboratory-developed tests that are based on Sanger sequencing and are performed in large central testing laboratories (i.e., requiring specimen transport to centralized locations such as those managed by LabCorp or Quest in the United States) have been extensively validated and approved by clinical laboratory regulatory agencies.

Thermo Fisher Scientific manufactures the most commonly used ABI DNA sequencers used for HIVDR testing. These are automated Sanger sequencing instruments based on capillary electrophoresis and include several different instrument models (e.g., models 3130, 3500, 3730, SeqStudio). Support for the smaller scale 3130 instrument is being discontinued, leaving only the more expensive 3500 and 3730 models, which range from 8 to 96 capillaries, and the SeqStudio platform. The advantages of the Sanger sequencing approach include the one-to-one correspondence between a sequencing lane and a sample (i.e., no “barcodes” are required) and the wealth of experience with the platform. Read lengths of individual sequences can be up to ~800 bases, and the sensitivity for detection for low abundance variants is approximately 20%. Sanger sequencing is particularly suitable where low-to-medium throughput is required with relatively small batch sizes. This, as well as the relatively large pre-existing base of ThermoFisher/ABI sequencers in the field, experienced users, and the availability of software which can process their data has led to their usage in most of the LMICs with capacity to perform HIVDR testing. The World Health Organization (WHO) has published recommendations for sequencing assay validation and minimum requirements for laboratories that perform sequencing in support of HIVDR surveillance [29].

### “Next generation” sequencing approaches

“Next generation” and even “third generation” sequencing (“NGS”) has replaced Sanger sequencing in many laboratories for many pathogens, although usually not for HIVDR. NGS uses parallel testing of a large number of templates to produce a vast amount of sequencing data at a low cost per base obtained (but not necessarily per sample tested). Many individual samples can be tested simultaneously by pooling samples (usually using molecular “barcodes” to identify each sample)–for example as many as 1000 samples on a single MiSeq run [30]. The use of unique molecular identifiers (UMIs, also known as “primer IDs”) in addition to the sample barcodes at the reverse-transcription step allows for the accurate quantification of the proportion of each individual variant [30]. Several publicly available and web-based bioinformatics pipelines specifically designed for the analysis of HIVDR have been described, and a special journal supplement was recently dedicated to discussing these methods [31].

Scaling up testing to handle large numbers of samples at once on Illumina instruments is straightforward due to the large capacity of these machines. For example, NGS sequencing of the SARS-CoV-2 genome [32] demonstrated that it is technically feasible to produce millions of consensus sequences from a small viral genome in a short time. Theoretically, a single Illumina NovaSeq 6000 (capable of producing up to 20 billion sequencing reads in under 24 hours), coupled with automated sample handling and data processing could easily handle the entire world’s HIVDR sequencing needs. However, these large-scale instruments are extremely expensive and require considerable technical expertise to operate. Thus, this approach would be most useful in use cases where centralization–or *extreme* centralization—of testing is desired.

#### SENTOSA SQ

The SENTOSA SQ HIV-1 Genotyping Assay (Vela Diagnostics) can detect mutations in *HIV*-1 Group M RNA (Table 4). It is the only FDA-approved test currently on the market and is the first such test to use NGS for HIV DR [33–35]. This is a highly integrated and automated system which includes extraction, amplification, barcoding, and library construction using robotic pipetting, and sequencing based on a specialized Ion Torrent sequencer known as the Sentosa SQ301. The SENTOSA SQ assay has its own analysis pipeline, including a susceptibility report that uses three HIVDR interpretation algorithms (Stanford HIVdb, ANRS, and REGA). It has been used to sequence both subtype B and non-B viruses(personal communication), and HIV proviral DNA, but to date it has not been widely used in LMICs [33–35]. The HIV RNA resistance testing system is FDA approved (Version 1) and a version 2 will soon be submitted for European approval. Despite reported concerns about relatively high cost per sample, and turnaround time [35], resistance data can be used to inform individual patient care.

**Table 4:** Commercially available next-generation sequencing HIV drug resistance assays.

	Advanced Biological Laboratories (ABL)	Vela Diagnostics
Product Name	DeepChek Assay PROTEASE / REVERSE TRANSCRIPTASE and DeepChek Assay INTEGRASE Genotyping and Drug Resistance	SENTOSA SQ HIV-1 Genotyping Assay
Target region	PR codons 1–99, RT codons 1–320, IN codons 20–280	PR codons 1–99, RT codons 1–337, IN codons 1–288
HIV-1 subtypes	Major HIV-group M viruses	Major HIV-group M viruses
Sample types	Plasma	Plasma
Assay description	HIV genotyping assay for either PR-RT, IN, or co-receptor inhibitor resistance using Illumina-based sequencing. Also available as a whole genome HIV genotyping assay and possible to use proprietary based robotics.	Integrated workflow from extraction to data analysis, using robotic pipetting, and sequencing based on a specialized Ion Torrent sequencer known as the Sentosa SQ301
Extraction method	User-based	Integrated system
Time to results	~3–4 days	2 days
Core equipment	DeepChek Pipetting Robot Titanium & Illumina sequencing systems	Sentosa SX101 extraction instrument, Sentosa ST401 system library preparation and Sentosa SQ301 for sequencing
Technical skills required	High	Medium
Regulatory approval	CE-marked	FDA approved
Strength	Availability as a kit with proprietary-based sequence analysis pipeline and data management system.	Highly integrated and automated system from extraction to data analysis
Weakness	Requires high level of skills	High costs, long turn-around-time

#### DeepChek

Advanced Biological Laboratories (ABL) produces an HIV genotyping assay for PI, NRTI, NNRTI and INSTI resistance using Sanger technology as well as a separate assay using Illumina-based sequencing (Tables 3 and 4). These assays as well as the related software (“DeepChek”) are CE-marked to allow automated analysis and resistance interpretation of NGS or Sanger data. More recently, a whole genome sequencing method for HIV was described using a scaled-down Illumina sequencer [36]. Although it is not clear that the entire HIV genome needs to be sequenced for current ARVs (or how to interpret mutations outside the target regions), the development of ARVs that target regions outside of *pol* may make this approach attractive in the future.

#### Lab-developed NGS tests

*Illumina sequencing*. The majority of NGS for HIVDR genotyping has been performed using laboratory-developed assays, especially on the mid-scale Illumina MiSeq model (~25 million reads per run). These are massively parallel, short-read (e.g., 150 bp) sequencing devices. The millions of generated reads require complex bioinformatic pipelines to assess, filter, map and align into a final consensus sequence. Illumina-based NGS can detect HIV variants present in low abundance, which is likely most relevant for characterizing NNRTI resistance [37]. While good concordance has been demonstrated between Illumina and Sanger sequencing for the most abundant variants in a given sample, at the lower ranges of variant abundance (e.g. below 5%), processing of the same raw data by three different software packages can lead to discordant results [38].

While Illumina sequencers may account for the vast majority of DNA sequencing data generated worldwide, they have had less of an impact on HIVDR genotyping as a routine test, primarily because the short length of HIV requires relatively little actual sequencing. Scaling the technology down to sequence a small number of samples in a cost-effective manner has been problematic. The Illumina sequencer is coupled with a large demand for bioinformatics processing (not yet standardized) and data storage which can further complicate its applicability in LMICs. There are also significant issues with training, logistics, standardized quality control and other practical issues. Illumina does not appear to have plans to develop its own HIV DR testing kit.

*Ion torrent*. The Ion Torrent sequencing platform (ThermoFisher) works on a different principle than Illumina sequencers, but also provides massively parallel sequencing capacity. In this approach, barcoded PCR product libraries are clonally amplified on capture beads using “emulsion PCR” to generate single-template substrates that are used for sequencing on semiconductor chips. Recently, a new assay has been described using this platform which includes not only the PCR product generation but also includes a freely available “end-to-end” analysis and reporting software [39]. A total of 17 PCR amplicons are produced in two independent amplification pools, allowing redundant coverage of almost every major resistance-associated position in HIV PR, RT, and IN. This combination of wet-lab, sequencing hardware and software was shown to have greater than 98% sensitivity and specificity relative to the ViroSeq Sanger-based comparator [39]. The sequences of the primers used were not disclosed by the authors, but they can be obtained from ThermoFisher.

*Pacific biosciences*. Pacific Biosciences (“PacBio”) makes DNA sequencers that use a novel technology enabling it to sequence long strands of DNA—for example the entire HIV genome—in a single read [40]. This approach could be of use particularly where mutation linkage information is required, if resistance to drugs other than PIs, NRTIs, NNRTIs or INSTIs are of interest, or if one is investigating contributions of regions outside of *pol* to HIVDR. Initial attempts to use this platform for HIV DR tests showed the individual reads from early PacBio systems were relatively inaccurate [41]. Recent improvements including the Single Molecule, Real-Time (SMRT) Sequencing and the Sequel II System allow >99% single molecule read accuracy, while still providing long reads. This platform demonstrated an ability to discern low-abundance variants in the HIV *pol* gene [42]. The platform is currently not widely used for HIVDR testing in LMICs in part because of its relatively high instrument costs (>$500,000). This cost may restrict its implementation to well-resourced countries, and to very centralized facilities, since (as for the Illumina platform) the high capital investment can be offset by the large amount of data which can be produced. Pacific Biosciences might develop its own HIVDR testing kit, although this plan is still to be confirmed (personal communication).

A laboratory-developed HIVDR test called HIV incidence and drug resistance assay (HIDA) was recently described by researchers at the University of Southern California. HIDA tags individual reverse-transcribed molecules with a “barcode” or “Unique Molecular Index” (UMI), followed by amplification of HIV *pol* or *env* sequences segregated into microdroplet compartments and then separately amplified and sequenced using long read sequencing [43]. These long-read sequences were obtained using SMRT sequencing on the PacBio Sequel II system. The detection limit for low abundance variants was reported to range between 2.5–10%, and linkage between mutations on the same genome could be determined. The advantages of this approach include the ability to accurately quantify individual variants using UMIs, coupled with long individual sequence reads. The data generated for diversity of variants in a sample can also be used as an indicator of the duration of infection [43], which may be useful in epidemiological surveillance and incidence studies. The assay is being commercialized, with the potential for future regulatory approval. The high equipment costs required for the assay (PacBio sequencing machine in addition to a BioRad QX20 microdroplet generator) suggest that it would be more appropriate for a centralized testing approach.

*Oxford nanopore*. Oxford Nanopore Technologies have both large and small, portable sequencing systems available. The smaller, palm-sized “MinIon” sequencer has a low upfront cost (~$5000) and is marketed to run on any desktop or laptop computer. This mobile approach has been used in tracking the Ebola outbreak in West Africa [44], and more recently in the COVID-19 pandemic [45].

In nanopore sequencing, a nucleic acid library is prepared using one of several kits in which a proprietary adaptor is ligated onto the nucleic acid molecules. The library is then loaded onto a flow cell array containing an electro-resistance membrane with embedded protein nanopores. An electrical current is applied across the flow cell array, and the electric current for each pore is recorded using a series of channels and sensor chips under conditions where nucleic acids pass through the nanopores at a defined rate. As the nucleic acids pass through the pore, an electrical current is disrupted in a sequence-specific manner which can be decoded to determine the DNA or RNA sequence.

One advantage of nanopore sequencing technology is the ability to sequence in real time. Within minutes of loading a nucleic acid library onto the sequencer, molecules are analyzed and can be resolved into individual reads. Bioinformatic software can be initialized to filter, map and align individual reads to a target reference for real-time detection of a pathogen such as HIV-1 or SARS-CoV-2. When combined with the portability of the sequencing device, point of care (POC) or near-POC HIVDR testing is possible. The length of sequencing reads can also be adjusted to balance data throughput against other factors including data storage and utility. While the portability of the MinIon (and smaller scale options in development) make this an attractive option for small scale sequencing, the company also supplies larger scale equipment (such as the Promethion) which would be more appropriate for centralized testing.

Nanopore technology still faces several challenges and limitations to its widespread adoption. Wide variation in sequencing accuracy particularly in regions with homopolymeric sequences and error rates have limited its use in the past. Given that some of the most relevant resistance-associated mutations for HIV are in homopolymeric regions (e.g., K65R and K103N in RT), this limitation appeared to disqualify nanopore sequencing as a viable method for HIVDR testing. However, continued improvements in the technology have been reported to result in higher accuracy, and newer approaches to further improve the homopolymeric sequence accuracy may improve the utility of this technology for HIVDR testing. Accuracy up to 99.6% (mode) for individual reads have been reported [46]. In addition, the company supplies a “field sequencing kit”, with many of the reagents supplied in lyophilized form that are stable at ambient temperatures for extended periods. The stability of the components makes it ideal for use in settings where access to cold storage may be limited.

Despite the clear advantages of the portability of the device, basecalling of raw nanopore data remains computationally expensive and requires a powerful graphics processing unit to ensure completion of basecalling by the end of a sequencing run. Most laptop computers lack the processing power to ensure timely basecalling, and Oxford Nanopores’ own portable Mk1C MinIon device may still require hours or even days after sequencing itself has completed, depending on how much data is collected. Simplified, complete bioinformatic processing of nanopore data for HIVDR testing would be required for broad uptake [47].

### Point mutation assays

Several HIVDR kits that are not based on sequencing are also in development. Most of these kits are potentially deployable at or near the POC, which would be extremely useful in settings where access to standard genotypic testing is limited. Such a test would be particularly useful in conjunction with or as a reflex from POC HIV viral load tests [48], especially if minimal sample handling were required and rapid turnaround possible. None of the point mutation assays described below have been approved for clinical use by US FDA or CE authorities.

#### Oligonucleotide ligation assay (OLA) and vOLA

OLA is a point mutation detection assay based on selective oligonucleotide ligation developed by researchers at the University of Washington [49]. The OLA-simple kit was developed to detect mutations associated with resistance to certain NRTIs and NNRTIs in HIV-1 subtypes A, B, C, D, and CRF01_AE (Table 5). The kit uses dried reagents to facilitate assay setup, lateral flow devices for visual detection, and in-house software with an interface for guiding analysis of results [49]. This test has been investigated in field studies in Kenya [50] and Mexico [51]. A newer iteration of OLA under development (“vOLA”) includes a POC viral load assay coupled with a reflex option for the detection of DR variants (personal communication).

**Table 5:** Potential commercially available point of care HIV drug resistance assays.

	Aldatu Biosciences	University of Washington
Product Name	PANDAA	OLA
Target region	K65R, K103N, V106M, Y181C, M184V/I and G190A	K65R, K103N, V106M/I, Y181C, M184V, and G190A
HIV-1 subtypes	Major HIV-group M	Major HIV-group M
Sample types	Plasma	Plasma
Assay description	A real-time, allele-specific PCR based assay that incorporates degenerate primers with fixed-sequence adaptor regions that overlap with the probe-binding site to account for the fact that viral sequences surrounding the target mutations being queried can also vary	Template dependent ligation of 2 primers and detection by ELISA. The kit uses dried reagents to facilitate assay setup, lateral flow devices for visual detection, and in-house software with an interface for guiding analysis of results
Extraction method	User-based	User-based
Time to results	~1–2 hours	6–8 hours
Core equipment	Quantitative real-time PCR equipment	Plate spectrophotometer
Technical skills required	Low-medium	Low-medium
Regulatory approval	None	None
Strength	Simplified workflow using readily available real-time PCR equipment	Sensitive, rapid, and affordable, set to be combined with a viral load POC assay (“vOLA”) coupled with a reflex option for the detection of DR variants which increases its usability in remote settings
Weakness	Currently includes only six mutation sites in PR and RT (not IN)	Only few mutations in PR and RT (not IN), specificity, affected by polymorphisms, low amplification sensitivity in non-B subtypes
Availability	Available for research use only	Still in development

#### Pan Degenerate Amplification and Adaptation (PANDAA) qDx HIVDR RTI

The PANDAA qDx HIVDR RTI resistance testing kit from Aldatu Biosciences (Boston, MA) is a real-time, allele-specific PCR based assay which detects the K65R, K103N, V106M, Y181C, M184V/I and G190A mutations in the RT region of the *pol* gene (Table 5). The PANDAA assay incorporates degenerate primers with fixed-sequence adaptor regions that overlap with the probe-binding site to account for the fact that viral sequences surrounding the target mutations being queried can also vary [52]. These variations greatly complicate the design of traditional allele-specific PCR assays. The kit design is based on a batch size of 96 samples (including controls) using a real-time quantitative PCR machine, and therefore may be best suited to centralized laboratory testing facilities, especially where these instruments already exist. The advantages of this assay are speed (1–2 hours), a relatively simple and familiar procedure for experienced laboratory technicians, and an automated analysis step. The target viral load ranges from 2000 to 750,000 HIV RNA copies/mL. The speed advantage is somewhat negated by the need to transfer samples to a centralized laboratory, but the simplified workflow is a distinct advantage. Versions of the PANDAA assay have been used in both treatment naïve [53] and treatment-experienced individuals [53, 54], and a recent comparison in Zimbabwe [53] and Botswana [55] showed good agreement with Sanger sequencing results. PANDAA qDx HIVDR RTI is available for RUO applications.

#### ANRS near point of care

The ANRS near POC diagnostic test [56] was developed at the University of Montpellier and combines sequence-specific primer extension with a lateral flow DNA microarray strip, allowing visual detection of HIVDR mutations in a short turnaround time (< 5 hours). To date, there has been a “proof of concept” study published on this approach, examining two NNRTI resistance associated mutations K103N and Y181C [56]. The visual detection step does not require specialized equipment, which is an advantage, but does seem to introduce a level of subjectivity.

#### Insilixa

Insilixa provides a platform for the large-scale identification of variant sequences using an array of surface-bound oligonucleotide probe sets, with a solid-phase melting curve analysis used to distinguish bound variants. In one study of different NNRTI mutations at HIV RT codon 103, it was able to detect low-abundance variants present at a proportion of ≥10% [57]. A particular strength of this platform is the ability to multiplex up to several hundred probes at a time (to account for the large amount of HIV sequence variation surrounding drug resistance sites both within and across HIV subtypes) and can still support POC deployment. In addition to detecting resistance this approach could potentially also simultaneously perform HIV viral load assessments [57]. This approach could have particular advantages where complete sequence data are not required.

In addition to the assays listed above, there are other approaches to detect DR-associated mutations which have been proposed, but whose current status is unknown or no longer under active development. These include a heteroduplex mobility assay [58], MALDI-TOF [59] and an eclectic range of different allele-specific PCR approaches.

**Fig 2:**
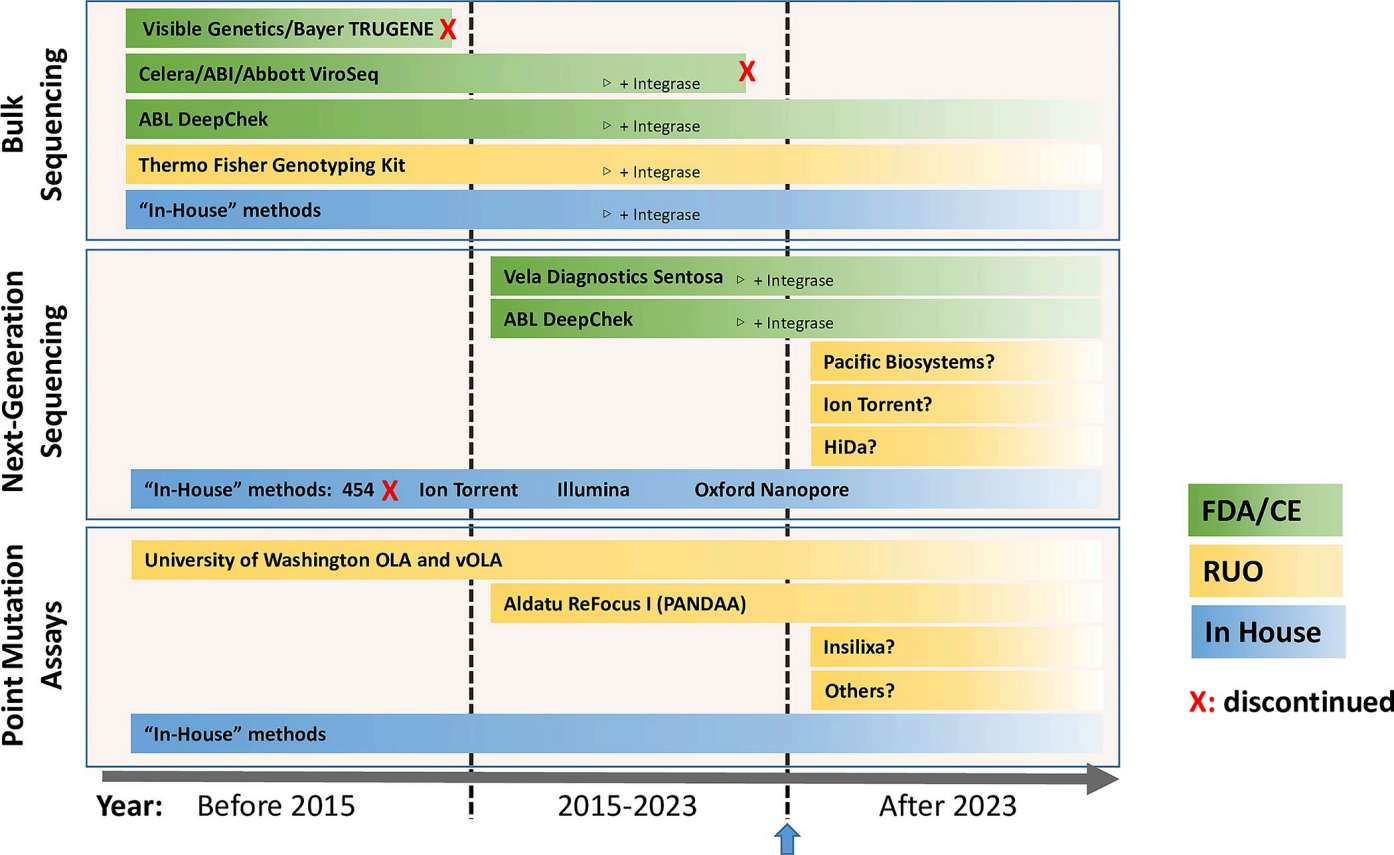
High-level timeline of the development and availability of HIV drug resistance tests. OLA: Oligonucleotide ligation assay. PANDAA: Pan-Degenerate Amplification and Adaptation. RUO: research use only. FDA: Food and Drug Administration. CE: conformité européenne.

An overview of the timeline of development and availability of all the HIV DR testing methods described above is presented in Fig 2.

## Discussion

This review summarises the landscape of technologies available for HIVDR testing for PIs, RTIs, and INSTIs. As indicated in Tables 1 and 2, there are approximately half a million individuals in LMICs who are expected to meet criteria for HIVDR every year according to WHO-defined Use Case 1 (after failure of a DTG-containing regimen) or Use Case 2 (after failure of multiple regimens including PI/r-based ART). In the future, additional use cases for HIVDR testing may be defined as ART treatment and prevention recommendations evolve. For example, the possibility of the development of INSTI resistance in the rare cases of HIV infection in people using long-acting cabotegravir for pre-exposure prophylaxis [60–62] could increase the forecasted number of DR tests that include integrase.

No current testing method can be considered ideal in every circumstance for meeting these use cases. The increasing usage of INSTI based ART in many LMICs has resulted in the decreased utility of assays assessing exclusively NRTI and/or NNRTI inhibitor resistance. Sanger-based sequencing is currently the most widespread technology in LMICs, but it is expected to be eventually replaced by NGS methods. These newer methods could be centralized and scaled up, but currently there is a lack of the required (expensive) equipment, expertise, and supply chains in LMICs, making this a challenging approach for resistance testing in many countries. The Oxford Nanopore system could potentially be used in a more mobile, scaled-down fashion, but currently requires more optimization to be used for routine HIVDR testing.

The number of test kits which could be used in clinical centres worldwide that are approved by main regulatory bodies (such as the US FDA and CE-IVD) is relatively small, and two of the previously available, approved kits have been discontinued. Such tests are more expensive than laboratory-developed tests, limiting their use in LMICs. However, it is important to note that the use of laboratory-developed tests for surveillance or patient management requires significant time and expense for validation, documentation, troubleshooting, batch validation, assay development and ongoing quality control. Therefore, reported costs per sample for laboratory-developed assays cannot be directly compared to kit-based test costs. Point mutation assays also apply genotypic approaches but have the inherent limitation that they can only detect known resistance-associated mutations, mostly related to NRTIs and NNRTIs. As most countries have or are transitioning to INSTIs, this constraint limits their use to fewer settings.

Finally, it is worth considering that some mutations which may be relevant for resistance may not be detected by any of the testing methods currently available if these lie outside the *pol* gene. Furthermore, there are other ARV drugs either available or soon to be available–including fostemsavir, lenacapavir and others—which have entirely different mechanisms of action and for which there are no widespread testing kits or protocols. These other drugs are certainly out of scope for most current global use cases, but this could change in the future.

## Conclusions

This review has informed the WHO’s process of developing a Target Product Profile (TPP) for HIVDR tests for use in individual patients. The aim of the TPP is to guide diagnostic product development efforts with regard to essential (minimal) and optimal product requirements, and can ensure alignment of objectives across stakeholders, accelerate development timelines, minimize development risks, and eventually lead to an optimal product that meets user needs in different situations. Ideal HIVDR tests would require simpler and cheaper sample collection and storage matrices, simplified sample processing, heat-stable reagents, simpler sequencing, workflows which capture PR, RT, and IN (or the whole genome) without additional labor. These would require seamless data analyses, and cost-effectively scale from testing a few samples at a time to testing many thousands at once.

Despite the availability of several HIVDR testing methods, their use in LMICs is limited by financial and programmatic constraints, including lack of expertise on the use and interpretation of the test, and in some instances by the limited applicability in a changing treatment landscape. More research and investment are needed to develop a HIVDR test meeting WHO specifications and adapted to LMICs.
